# ANXA7 promotes the cell cycle, proliferation and cell adhesion-mediated drug resistance of multiple myeloma cells by up-regulating CDC5L

**DOI:** 10.18632/aging.103326

**Published:** 2020-06-10

**Authors:** Haiyan Liu, Dan Guo, Yuou Sha, Chenlu Zhang, Yijing Jiang, Lemin Hong, Jie Zhang, Yuwen Jiang, Ling Lu, Hongming Huang

**Affiliations:** 1 Department of Hematology, The Affiliated Hospital of Nantong University, Nantong 226001, China; 2 School of Basic Medicine, Tongji Medical College, Huazhong University of Science and Technology, Wuhan 430030, China

**Keywords:** ANXA7, CDC5L, multiple myeloma, cell cycle, drug resistance

## Abstract

This study aimed to investigate whether annexin A7 (ANXA7) could promote the cell cycle, proliferation and cell adhesion-mediated drug resistance (CAM-DR) of multiple myeloma (MM) cells by up-regulating cell division cycle 5-like (CDC5L). As a result, ANXA7 expression was increased in the serum of MM patients and the expression of ANXA7 and CDC5L was also increased in MM cell lines. ANXA7 overexpression promoted the proliferation and cycle of U266 and RPMI8226 cells. The expression of proliferation cell nuclear antigen (PCNA), KI67, cyclin dependent kinase 1 (CDK1) and cyclinB1 in transfected cells was consistent with the changes of proliferation and cell cycle. In co-culture system of BMSC cells and MM cells, expression of CD44, ICAM1 and VCAM1 in MM cells was increased, which was further increased by ANXA7 overexpression. Bortezomib could increase the apoptosis of U266 and RPMI8226 cells. In co-culture system of BMSC cells and MM cells, the promotion effects of bortezomib on apoptosis of MM cells was decreased, which was further suppressed by ANXA7 overexpression. The above effects exerted by ANXA7 overexpression could be reversed by ANXA7 interference. Moreover, ANXA7 was proved to be combined with CDC5L. CDC5L interference could inhibit the promotion effects of ANXA7 overexpression on proliferation and cell cycle and inhibition effects of ANXA7 overexpression on apoptosis of MM cells treated with bortezomib in co-culture system. In conclusion, ANXA7 could promote the cell cycle, proliferation and CAM-DR of MM cells by up-regulating CDC5L.

## INTRODUCTION

Multiple myeloma (MM) is a widespread and incurable disease caused by the malignant proliferation and abnormal accumulation of clonal marrow plasma cells [1]. Most of them are middle-aged and elderly patients, with an average age of about 69 years and an average survival of 4-6 years. The incidence rate is 1/100,000. In recent years, MM incidence has been increasing year by year and the age of onset has become younger, accounting for about 13% of hematological malignancies and 1% of all malignancies [2, 3]. To date, most clinical treatments for MM have been chemoradiotherapy, autologous/allogeneic stem cell transplantation and targeted drug therapy to improve the quality of life and prolong the survival of patients, but the occurrence of acquired drug resistance makes MM still incurable, which has become one of the biggest challenges for MM [4–6]. Therefore, in order to bring new hope to MM patients, we must work harder to study the complex pathogenesis of MM and find more appropriate therapies for early diagnosis of MM.

Different members of the Annexin family are located on different intracellular biofilms and play important roles in the cytoskeleton activity, cell membrane phospholipid, cell adhesion, membrane receptor regulation, membrane transport and mitosis [7, 8]. Annexin A7 (ANXA7) is an important member of the Annexin family. Studies have shown that ANXA7 has Ca^2+^ dependent membrane fusion activity and can promote membrane fusion, adhesion and transport [9, 10]. Meanwhile, ANXA7 can also mediate the Ca^2+^/GTP signaling pathway by stimulating GTPase [11]. Membrane-linked protein A7 (ANXA7) is not consistently expressed in different types of cancer. Study showed that ANXA7 inhibition suppressed the growth of gastric cancer cells in vitro and in vivo and promote their apoptosis [12]. In hepatocellular carcinoma (HCC), ANXA7 silencing inhibited the proliferation and migration of HCC through the MAPK/ERK signaling pathway [13]. ANXA7 is an inhibitor of the occurrence and metastasis of prostate cancer [14]. However, ANXA7 expression in MM cells remains unknown. Cancer cell line encyclopedia (https://portals.broadinstitute.org/ccle/) predicts that ANXA7 expression is up-regulated in MM cells. Therefore, the effect of ANXA7 on MM needs to be further explored.

Cell division cycle 5-like (CDC5L) is a cell cycle regulatory element of G2/M transformation and is involved in the catalytic steps of mRNA splicing and DNA damage repair. Studies indicated that CDC5L expression in glioma and hepatocellular carcinoma was increased, and CDC5L interference could increase the cell cycle arrest in G2 phase and inhibit the proliferation of glioma cells and hepatoma cells [15, 16]. However, CDC5L has not been studied in MM. Cancer cell line encyclopedia (https://portals.broadinstitute.org/ccle/) predicts that CDC5L expression is increased in MM cell lines. Hence, what the role of CDC5L in MM is worth studying.

The string database predicts that ANXA7 can combine with CDC5L. Therefore, we further hypothesized that ANXA7 interference could promote cell cycle arrest in G2/M phase through CDC5L to inhibit proliferation of MM cells and reduce cell adhesion-mediated drug resistance (CAM-DR).

## RESULTS

### ANXA7 expression is increased in the serum of MM patients and MM cell lines

**Figure 1:**
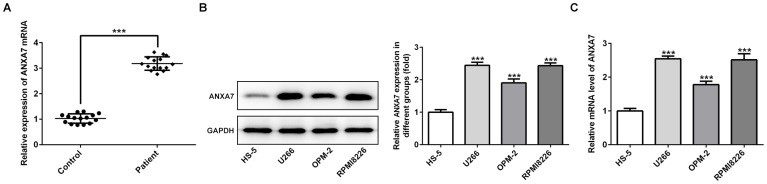
**ANXA7 expression is increased in the serum of MM patients and MM cell lines.** (**A**) The ANXA7 expression in the serum of MM patients was detected by RT-qPCR analysis. ***P<0.001 vs. Control group. (**B**) The mRNA expression of ANXA7 in MM cell lines was detected by RT-qPCR analysis. ***P<0.001 vs. HS-5 group. (**C**) The protein expression of ANXA7 in MM cell lines was detected by Western blot analysis. ***P<0.001 vs. HS-5 group.

The mRNA expression of ANXA7 was up-regulated in the serum of MM patients compared with that in healthy donors (Figure 1A). As shown in Figure 1B and 1C, the mRNA expression and protein expression of ANXA7 was increased in U266, OPM-2 and RPMI-8226 cells compared with HS-5 cells. U266 and PRMI-8266 cells with high expression of ANXA7 were selected for the following experimental study.

### ANXA7 overexpression promotes the proliferation of U266 and RPMI8226 cells

**Figure 2:**
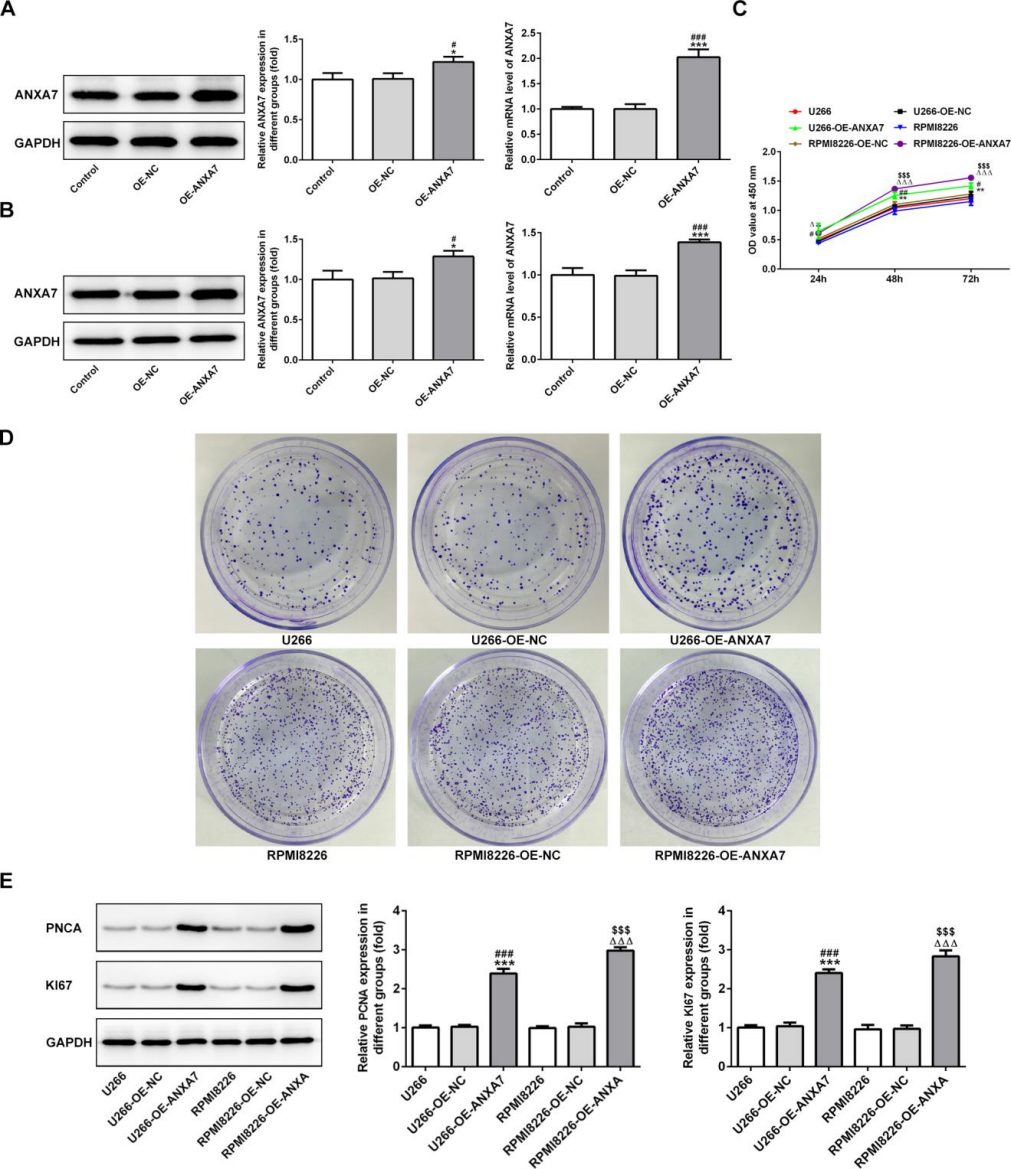
**ANXA7 overexpression promotes the proliferation of U266 and RPMI8226 cells.** (**A**) The expression of ANXA7 in U266 cells after transfection of OE-ANXA7 was detected by Western blot and RT-qPCR analysis. *P<0.05 and ***P<0.001 vs. Control group. ^#^P<0.05 and ^###^P<0.001 vs. OE-NC group. (**B**) The expression of ANXA7 in RPMI8226 cells after transfection of OE-ANXA7 was detected by Western blot and RT-qPCR analysis. *P<0.05 and ***P<0.001 vs. Control group. ^#^P<0.05 and ^###^P<0.001 vs. OE-NC group. (**C**) The proliferation of U266 and RPMI8226 cells after transfection of OE-ANXA7 was determined by CCK-8 assay. **P<0.01 vs.U266 group. ^#^P<0.05 and ^##^P<0.01 vs. U266+OE-NC group. ^ΔΔΔ^P<0.001 vs. RPMI8226 group. ^$$$^P<0.001 vs. RPMI8226-OE-NC group. (**D**) The proliferation of U266 and RPMI8226 cells after transfection of OE-ANXA7 was also showed by colony formation assay. (**E**) The protein expression of PCNA and KI67 in U266 and RPMI8226 cells after transfection of OE-ANXA7 was detected by Western blot analysis. ***P<0.001 vs.U266 group. ^###^P<0.001 vs. U266-OE-NC group. ^ΔΔΔ^P<0.001 vs. RPMI8226 group. ^$$$^P<0.001 vs. RPMI8226-OE-NC group.

After transfection, ANXA7 expression was increased in both U266 and RPMI8226 cells when they were transfected with overexpression-ANXA7 compared with control group and OE-NC group (Figure 2A and 2B). ANXA7 overexpression promoted the proliferation of U266 and RPMI8226 cells (Figure 2C), which also demonstrated by colony formation assay (Figure 2D). The result of Figure 2E indicated that the protein expression of PCNA and KI67 was increased in OE-ANXA7 transfected U266 and RPMI8226 cells.

### ANXA7 overexpression accelerates the cycle of U266 and RPMI8226 cells

**Figure 3:**
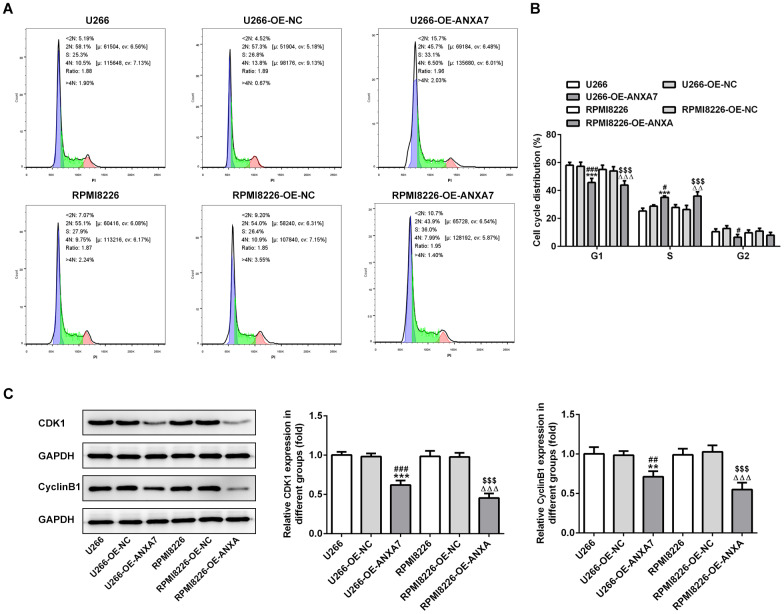
**ANXA7 overexpression accelerates the cycle of U266 and RPMI8226 cells.** (**A**) The images of flow cytometry for U266 and RPMI8226 cells after transfection of OE-ANXA7. (**B**) The cell cycle distribution of U266 and RPMI8226 cells after transfection of OE-ANXA7 was analyzed by flow cytometry analysis. ***P<0.001 vs.U266 group. ^#^P<0.05 and ^###^P<0.001 vs. U266+OE-NC group. ^ΔΔΔ^P<0.001 vs. RPMI8226 group. ^$$$^P<0.001 vs. RPMI8226-NC group. (**C**) The protein expression of CDK1 and cyclinB1 in U266 and RPMI8226 cells after transfection of OE-ANXA7 was detected by Western blot analysis. **P<0.01 and ***P<0.001 vs.U266 group. ^##^P<0.01 and ^###^P<0.001 vs. U266-OE-NC group. ^ΔΔΔ^P<0.001 vs. RPMI8226 group. $$$P<0.001 vs. RPMI8226-OE-NC group.

After U266 and RPMI8226 cells transfected with OE-ANXA7, ANXA7 overexpression shortened the G0/G1 phase and G2/M phase while extended the S phase (Figure 3A and 3B). As shown in Figure 3C, the protein expression of CDK1 and cyclinB1 was decreased when U266 and RPMI8226 cells were transfected with overexpression-ANXA7.

### ANXA7 interference inhibits the proliferation of U266 and RPMI8226 cells

**Figure 4:**
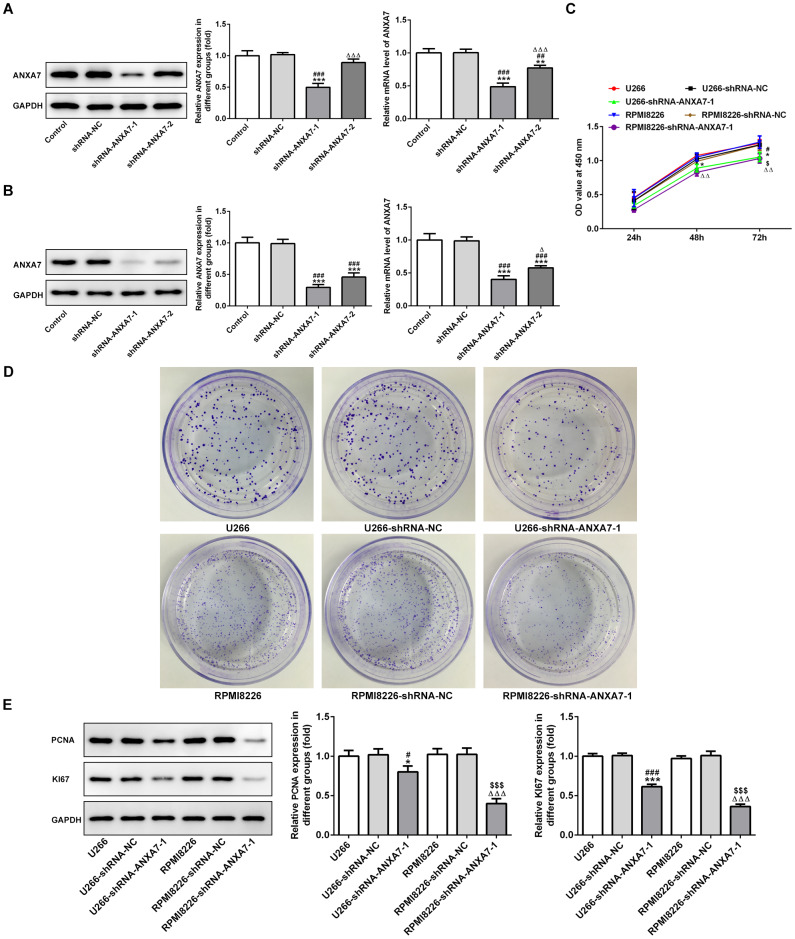
**ANXA7 interference inhibits the proliferation of U266 and RPMI8226 cells.** (**A**) The expression of ANXA7 in U266 cells after transfection of shRNA-ANXA7 was detected by Western blot and RT-qPCR analysis. **P<0.01 and ***P<0.001 vs. Control group. ^##^P<0.01 and ^###^P<0.001 vs. shRNA-NC group. ^ΔΔΔ^P<0.001 vs. shRNA-ANXA7-1 group. (**B**) The expression of ANXA7 in RPMI8226 cells after transfection of shRNA- ANXA7 was detected by Western blot and RT-qPCR analysis. ***P<0.001 vs. Control group. ^###^P<0.001 vs. shRNA-NC group. ^Δ^P<0.05 vs. shRNA-ANXA7-1 group. (**C**) The proliferation of U266 and RPMI8226 cells after transfection of shRNA- ANXA7 was determined by CCK-8 assay. *P<0.05 vs.U266 group. ^#^P<0.05 vs. U266-shRNA-NC group. ^ΔΔ^P<0.01 vs. RPMI8226 group. ^$^P<0.05 vs. RPMI8226-shRNA-NC group. (**D**) The proliferation of U266 and RPMI8226 cells after transfection of shRNA- ANXA7 was also showed by colony formation assay. (**E**) The protein expression of PCNA and KI67 in U266 and RPMI8226 cells after transfection of shRNA-ANXA7 was detected by Western blot analysis. ***P<0.001 vs.U266 group. ^###^P<0.001 vs. U266-shRNA-NC group. ^ΔΔΔ^P<0.001 vs. RPMI8226 group. ^$$$^P<0.001 vs. RPMI8226-shRNA-NC group.

As shown in Figure 4A and 4B, the protein expression of ANXA7 was decreased in U266 and RPMI8226 cells after transfection of shRNA-ANXA7-1 and shRNA-ANXA7-2 compared with shRNA-NC group. The protein expression of ANXA7 in shRNA-ANXA7-1 transfected cells was lower than that in shRNA-ANXA7-2 transfected cells. Therefore, shRNA-ANXA7-1 was chosen for the next experiment. CCK-8 assay indicated that the proliferation of U266 and RPMI8226 cells was inhibited by ANXA7 interference compared with that in shRNA-NC transfected cells (Figure 4C). The result of colony formation assay was the same with the result of CCK-8 assay (Figure 4D). As shown in Figure 4E, the protein expression of PCNA and KI67 in U266 and RPMI8226 cells was suppressed by ANXA7 interference compared with that in shRNA-NC transfected cells.

### ANXA7 interference leads to G2/M arrest of cell cycle

**Figure 5:**
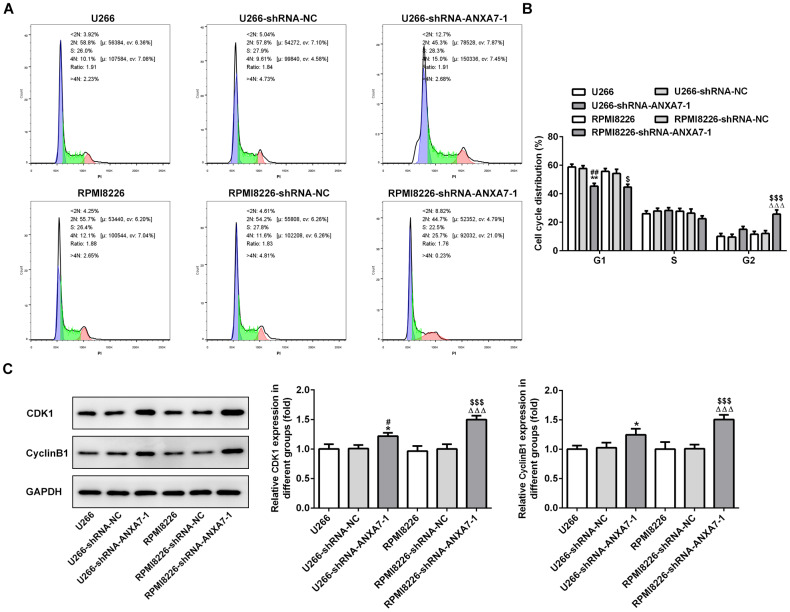
**ANXA7 interference leads to G2/M arrest of cell cycle.** (**A**) The images of flow cytometry for U266 and RPMI8226 cells after transfection. (**B**) The cell cycle distribution of U266 and RPMI8226 cells after transfection of shRNA-ANXA7 was analyzed by flow cytometry analysis. **P<0.01 vs.U266 group. ^##^P<0.01 vs. U266-shRNA-NC group. ^ΔΔΔ^P<0.001 vs. RPMI8226 group. ^$^P<0.05 and ^$$$^P<0.001 vs. RPMI8226-shRNA-NC group. (**C**) The protein expression of CDK1 and cyclinB1 in U266 and RPMI8226 cells after transfection of shRNA-ANXA7 was detected by Western blot analysis. *P<0.05 vs.U266 group. ^#^P<0.05 vs. U266-shRNA-NC group. ^ΔΔΔ^P<0.001 vs. RPMI8226 group. ^$$$^P<0.001 vs. RPMI8226-shRNA-NC group.

ANXA7 interference decreased the G0/G1 phase while increased the G2/M phase. The G0/G1 phase, S phase and G2/M phase were not obviously changed in shRNA-NC transfected cells compared with control (U266 and RPMI8226) group. The change of S phase after shRNA-ANXA7-1 transfection was not significant (Figure 5A and 5B). As shown in Figure 5C, the protein expression of CDK1 and cyclinB1 was up-regulated in U266 and RPMI8226 cells when they were transfected with shRNA-ANXA7-1 compared with that in shRNA-NC transfected cells.

### ANXA7 overexpression increases the cell adhesion-mediated drug resistance (CAM-DR), which was inhibited by ANXA7 interference

**Figure 6:**
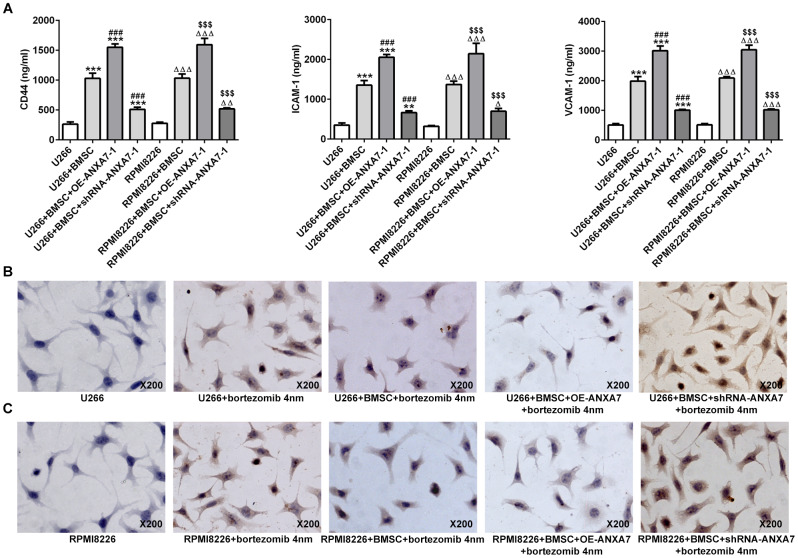
**ANXA7 overexpression increases the cell adhesion-mediated drug resistance (CAM-DR), which was inhibited by ANXA7 interference.** (**A**) The levels of CD44, ICAM1 and VCAM1 in U266 and RPMI8226 cells co-cultured with BMSC after transfection were detected by ELISA assay. **P<0.01 and ***P<0.001 vs.U266 group. ^###^P<0.001 vs. U266+BMSC group. ^ΔΔΔ^P<0.001 vs. RPMI8226 group. ^$$$^P<0.001 vs. RPMI8226+BMSC group. (**B**) The apoptosis of U266 cells treated with bortezomib in co-culture system was determined by TUNEL assay. (**C**) The apoptosis of RPMI8226 cells treated with bortezomib co-culture system was determined by TUNEL assay.

By co-culture of cell-cell contact with BMSC cells, the expression of CD44, ICAM1 and VCAM1 was all increased in U266 and RPMI8226 cells, which was further increased by ANXA7 overexpression and inhibited by ANXA7 interference (Figure 6A). When U266 and RPMI8226 cells were treated with bortezomib, the cell apoptosis was increased. However, by co-culture with BMSC cells, the apoptosis-promoting effect of bortezomib on U266 and RPMI8226 cells was weakened. Furthermore, ANXA7 overexpression further inhibited the apoptosis-promoting effect of bortezomib on U266 and RPMI8226 cells while ANXA7 interference made the cells more sensitive to bortezomib, thereby promoting the cell apoptosis (Figure 6B and 6C).

### ANXA7 can be combined with CDC5L

**Figure 7:**
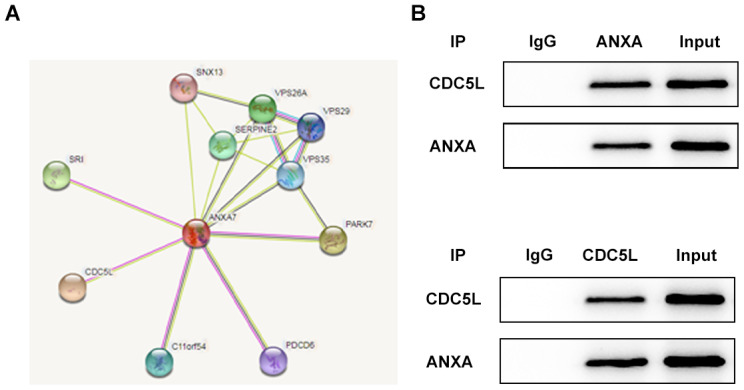
**ANXA7 can be combined with CDC5L.** (**A**) The string database predicts that ANXA7 can bind to CDC5L. (**B**) The combination of ANXA7 and CDC5L was determined by co-immunoprecipitation method. ***P<0.001 vs. IgG group. ^#^P<0.05 and ^##^P<0.01 vs. IP group.

The string database predicts that ANXA7 can bind to CDC5L and CDC5L is a cell cycle regulator protein associated with the G2/M phase (Figure 7A). As shown in Figure 7B, the protein expression of CDC5L was existed in anti-ANXA group and the protein expression of ANXA was existed in anti-CDC5L group, which showed that CDC5L was combined with ANXA.

### CDC5L expression is increased in multiple myeloma cell lines, which can be promoted by ANXA7

**Figure 8:**
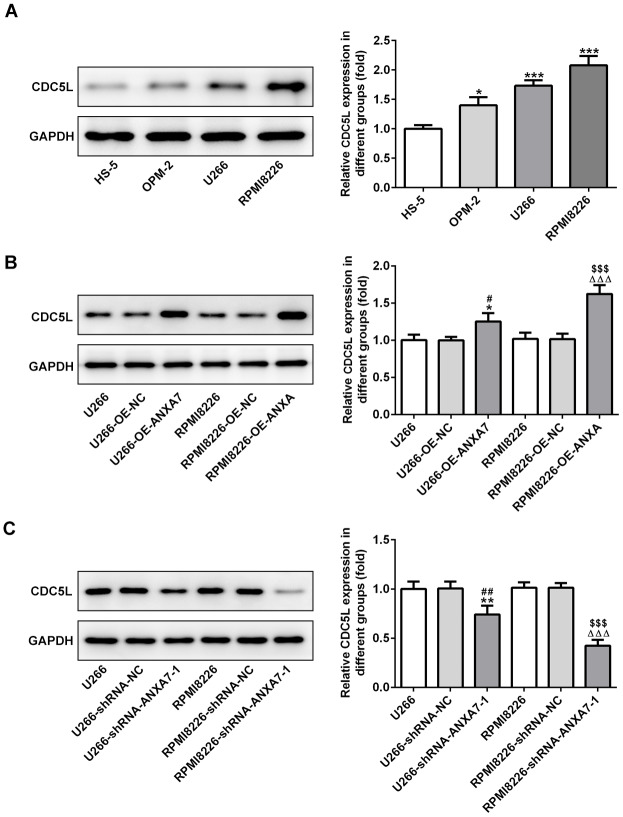
**CDC5L expression is increased in multiple myeloma cell lines, which can be promoted by ANXA7.** (**A**) The protein expression of CDC5L in MM cell lines was detected by Western blot analysis. *P<0.05 and ***P<0.001 vs. HS-5 group. (**B**) The protein expression of CDC5L in U266 and RPMI8226 cells after transfection of OE-ANXA7 was detected by Western blot analysis. *P<0.05 vs.U266 group. ^#^P<0.05 vs. U266-OE-NC group. ^ΔΔΔ^P<0.001 vs. RPMI8226 group. ^$$$^P<0.001 vs. RPMI8226- OE-ANXA7 group. (**C**) The protein expression of CDC5L in U266 and RPMI8226 cells after transfection of shRNA-ANXA7 was detected by Western blot analysis. **P<0.01 vs.U266 group. ^##^P<0.01 vs. U266-shRNA-NC group. ^ΔΔΔ^P<0.001 vs. RPMI8226 group. ^$$$^P<0.001 vs. RPMI8226-shRNA-ANXA7 group.

The protein expression of CDC5L was increased in U266, OPM-2 and RPMI-8226 cells compared with HS-5 cells (Figure 8A) and U266 and RPMI-8226 cells were selected for the subsequent experiment. When U266 and RPMI-8226 cells were transfected with OE-ANXA7, the protein expression of CDC5L was up-regulated in U266 and RPMI-8226 cells compared with OE-NC transfected cells (Figure 8B). When U266 and RPMI-8226 cells were transfected with shRNA-ANXA7-1, the expression of CDC5L was down-regulated in U266 and RPMI-8226 cells compared with OE-NC transfected cells (Figure 8C).

### CDC5L interference inhibits the proliferation promotion effect of ANXA7

**Figure 9:**
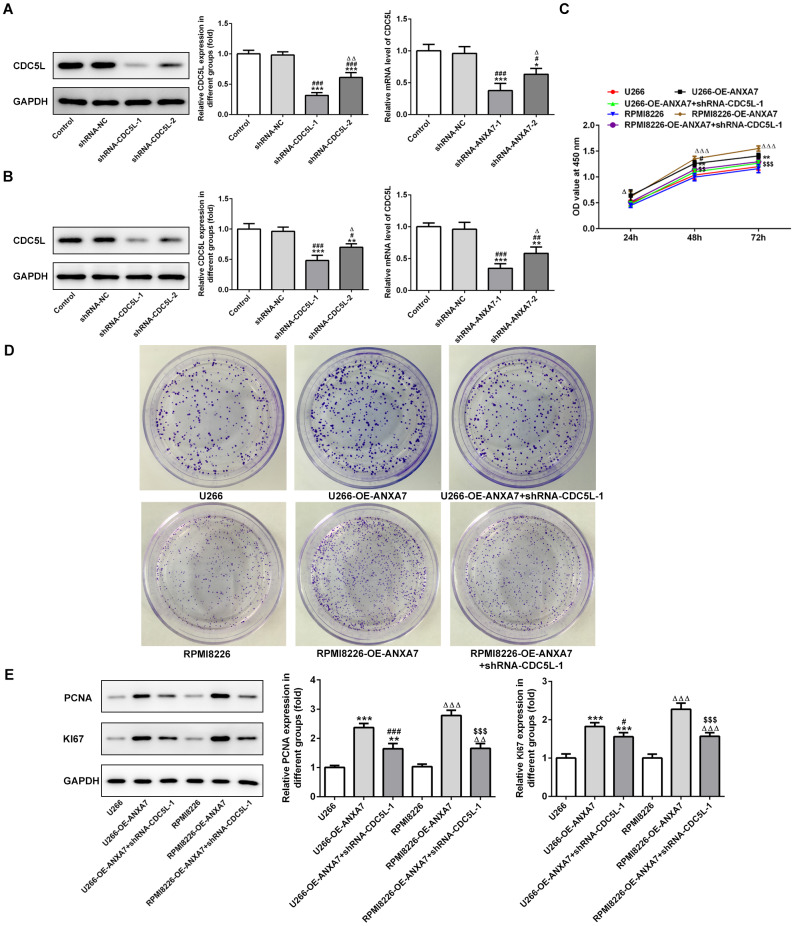
**CDC5L interference inhibits the proliferation promotion effect of ANXA7.** (**A**) The expression of CDC5L in U266 cells after transfection of shRNA-CDC5L was detected by Western blot and RT-qPCR analysis. *P<0.05 and ***P<0.001 vs. Control group. ^#^P<0.05 and ^###^P<0.001 vs. shRNA-NC group. ^Δ^P<0.05 and ^ΔΔ^P<0.01 vs. shRNA-CDC5L-1 group. (**B**) The expression of CDC5L in RPMI8226 cells after transfection of shRNA-CDC5L was detected by Western blot and RT-qPCR analysis. **P<0.01 and ***P<0.001 vs. Control group. ^#^P<0.05, ^##^P<0.01 and ^###^P<0.001 vs. shRNA-NC group. ^Δ^P<0.05 vs. shRNA-CDC5L-1 group. (**C**) The proliferation of U266 and RPMI8226 cells after transfection of shRNA-CDC5L was determined by CCK-8 assay. **P<0.01 vs.U266 group. ^#^P<0.05 vs. U266-OE-ANXA7 group. ^ΔΔΔ^P<0.001 vs. RPMI8226 group. ^$$^P<0.01 and ^$$$^P<0.001 vs. RPMI8226-OE-ANXA7 group. (**D**) The proliferation of U266 and RPMI8226 cells after transfection of shRNA-CDC5L was also showed by colony formation assay. (**E**) The protein expression of PCNA and KI67 in U266 and RPMI8226 cells after transfection of shRNA-CDC5L was detected by Western blot analysis. **P<0.01 and ***P<0.001 vs.U266 group. ^#^P<0.05 and ^###^P<0.05 vs. U266-OE-ANXA7 group. ^ΔΔ^P<0.01 and ^ΔΔΔ^P<0.001 vs. RPMI8226 group. ^$$$^P<0.001 vs. RPMI8226-OE-ANXA7 group.

After U266 and RPMI-8226 cells were transfected with shRNA-CDC5L-1 and shRNA- CDC5L-2, the protein expression of CDC5L was decreased. The protein expression of CDC5L in shRNA-CDC5L-1 group was lower than that in shRNA-CDC5L-2 group. Therefore, shRNA-CDC5L-1 was chosen for the subsequent experiment (Figure 9A and 9B). As shown in Figure 9C, ANXA7 overexpression promoted the proliferation activity of U266 and RPMI-8226 cells while CDC5L interference could inhibited the proliferation promotion effect of ANXA7 on U266 and RPMI-8226 cells, which confirmed by the result of colony formation assay (Figure 9D). The protein expression of proliferation related proteins (PCNA and KI67) was increased in U266 and RPMI-8226 cells transfected with OE-ANXA7. However, CDC5L interference reduced the protein expression of PCNA and KI67 in U266 and RPMI-8226 cells transfected with OE-ANXA7 (Figure 9E).

### CDC5L interference inhibits the cell cycle promotion effect of ANXA7

**Figure 10:**
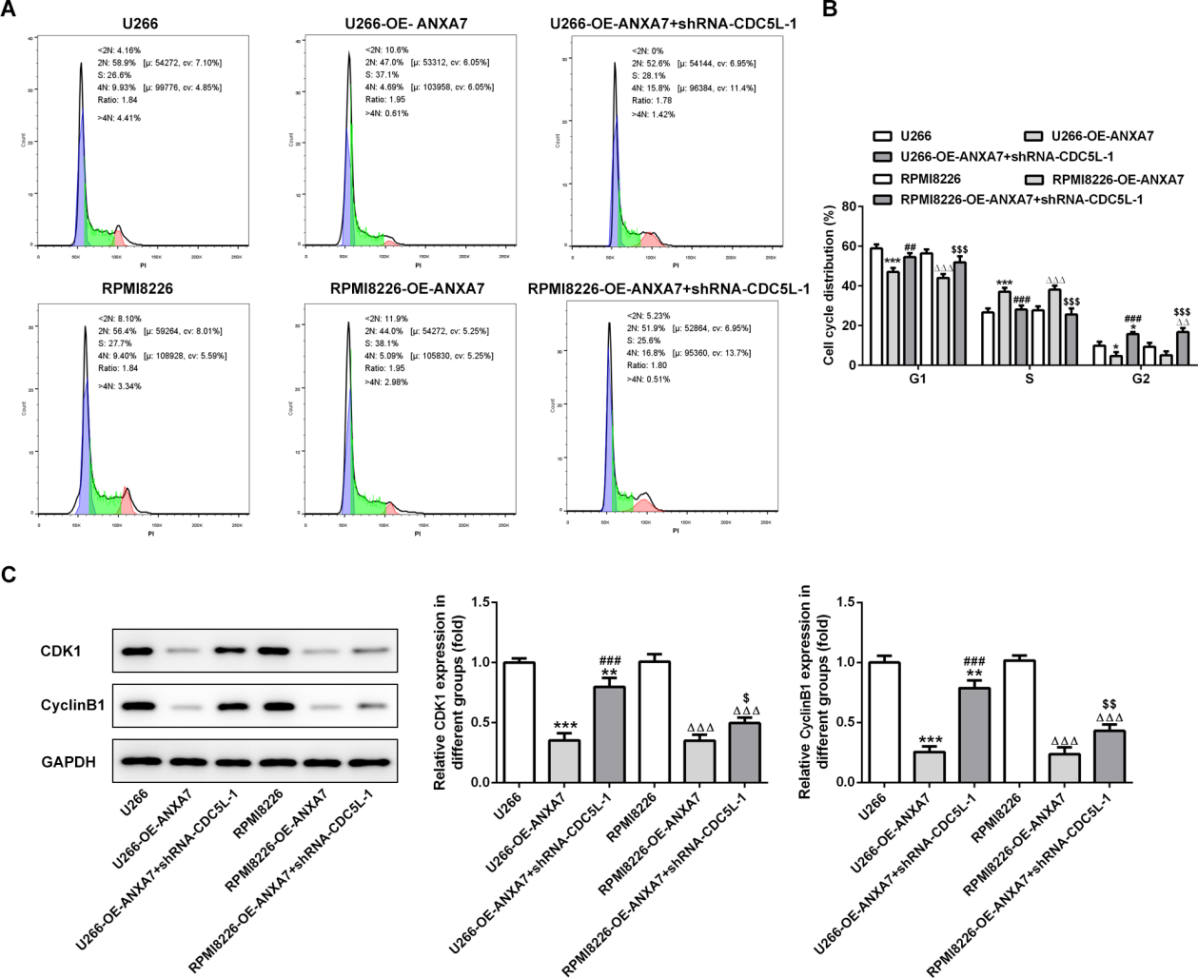
**CDC5L interference inhibits the cell cycle promotion effect of ANXA7.** (**A**) The images of flow cytometry for U266 and RPMI8226 cells after transfection of shRNA-CDC5L. (**B**) The cell cycle distribution of U266 and RPMI8226 cells after transfection of shRNA-CDC5L was analyzed by flow cytometry analysis. *P<0.05 and ***P<0.001 vs.U266 group. ^##^P<0.01 and ^###^P<0.001 vs. U266-OE-ANXA7 group. ^ΔΔ^P<0.01 and ^ΔΔΔ^P<0.001 vs. RPMI8226 group. ^$$$^P<0.001 vs. RPMI8226-OE-ANXA7 group. (**C**) The protein expression of CDK1 and cyclinB1 in U266 and RPMI8226 cells after transfection of shRNA-CDC5L was detected by Western blot analysis. **P<0.01 and ***P<0.001 vs.U266 group. ^###^P<0.001 vs. U266-OE-ANXA7 group. ^ΔΔΔ^P<0.001 vs. RPMI8226 group. ^$^P<0.05 and ^$$^P<0.01 vs. RPMI8226-OE-ANXA7 group.

ANXA7 overexpression decreased the G0/G1 phase and G2/M phase while increased S phase in OE-ANXA7 transfected U266 and RPMI-8226 cells. When U266 and RPMI-8226 cells were transfected with OE-ANXA7 and shRNA-CDC5L-1 simultaneously, CDC5L interference reversed the cell cycle promotion effect of ANXA7 on U266 and RPMI-8226 cells (Figure 10A and 10B). ANXA7 overexpression decreased the protein expression of cell cycle related proteins (CDK1 and cyclinB1) while CDC5L interference could raise the protein expression of CDK1 and cyclinB1 (Figure 10C).

### CDC5L interference inhibits the CAM-DR promotion effect of ANXA7

**Figure 11:**
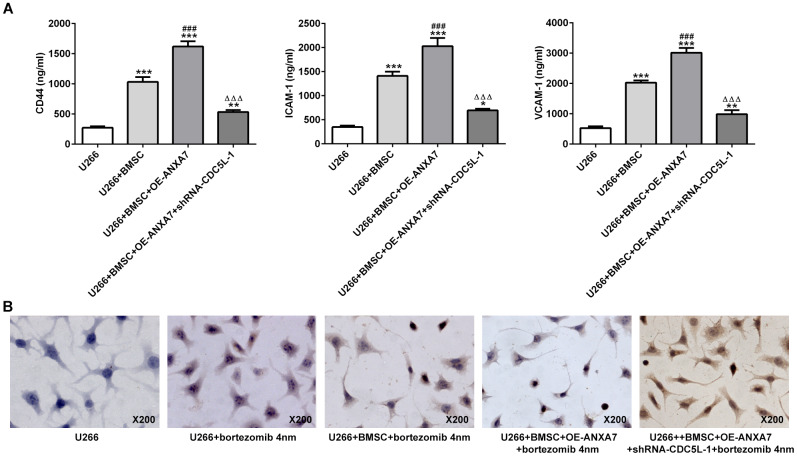
**CDC5L interference inhibits the CAM-DR promotion effect of ANXA7.** (**A**) The levels of CD44, ICAM1 and VCAM1 in U266 co-cultured with BMSC after transfection were detected by ELISA assay. *P<0.05, **P<0.01 and ***P<0.001 vs.U266 group. ^###^P<0.001 vs. U266+BMSC group. ^ΔΔΔ^P<0.001 vs. U266+BMSC+ OE-ANXA7 group. (**B**) The apoptosis of U266 cells treated with bortezomib in co-culture system was determined by TUNEL assay.

The expression of cell adhesion molecules (CD44, ICAM1 and VCAM1) was increased when U266 cells were co-cultured with BMSC cells. ANXA7 overexpression promoted the expression of CD44, ICAM1 and VCAM1 in co-cultured systems of U266 cells and BMSC cells. However, CDC5L interference reversed the promotion effects of ANXA7 on the expression of CD44, ICAM1 and VCAM1 in co-cultured system of U266 cells and BMSC cells (Figure 11A). The apoptosis of U266 cells was increased when U266 cells were treated with bortezomib. When U266 cells were co-cultured with BMSC cells, the cell apoptosis was decreased compared with U266+bortezomib group. ANXA7 overexpression further decreased the cell apoptosis in co-cultured system of U266 cells and BMSC cells. However, CDC5L interference increased cell apoptosis which reversed the CAM-DR promotion effect of ANXA7 on U266 cells (Figure 11B).

## DISCUSSION

Here, we aimed to investigate whether ANXA7 interference could promote cell cycle arrest in G2/M phase through CDC5L to inhibit proliferation of MM cells and reduce cell adhesion-mediated drug resistance. It was shown that ANXA7 interference could promote cell cycle arrest in G2/M phase through CDC5L to inhibit proliferation of MM cells and reduce cell adhesion-mediated drug resistance.

ANXA7 is involved in the membrane transporter, cell differentiation, apoptosis, growth regulation and calcium ion signaling pathways [17, 18]. Many studies have found ANXA7 expression was abnormal in a variety of tumor tissues [19]. The levels of ANXA7 expression in liver cancer, gastric cancer, nasopharyngeal cancer, colorectal cancer, cervical squamous cell carcinomas and breast cancer were increased [19–24]. In this study, ANXA7 expression was up-regulated in serum of MM patients and MM cells. Studies indicated that ANXA7 inhibition could inhibit the growth, proliferation and migration and promote apoptosis of cancer cells [12, 13]. This study presented that ANXA7 overexpression promoted the cell proliferation and cell cycle, which was reversed by ANXA7 inhibition.

CDC5L was involved in cell cycle regulation, and CDC5L inhibition in cells arrested the cell cycle at G2/M phase [25]. In bladder cancer, CDC5L expression was obviously increased and CDC5L expression was significantly related to pathology grade and Ki67 expression in bladder cancer. Furthermore, CDC5L inhibition suppressed proliferation, migration, invasion and EMT while induced apoptosis of bladder cancer cells [26]. Here, we have confirmed that CDC5L interference can effectively inhibit the promotion effects on proliferation and cell cycle induced by ANXA7 overexpression. At present, it has been found that tumor multiple drug resistance (MDR) may be related to the role of extracellular matrix (ECM) in the process of tumor cells treated with chemotherapy drugs. The susceptibility of tumor cells adhering to the ECM to a variety of chemotherapeutic drugs is significantly reduced and the drug resistance called cell adhesion-mediated drug resistance (CAM-DR). Oxorubicin, bortezomib, carfilzomib, and pomalidomide have emerged as effective agents to improve clinical outcomes for patients in all stages of MM. However, these drugs may lose their efficacy because of CAM-DR in refractory MM (RRMM) [27–32]. Study has shown that cell adhesion molecules and their mediated adhesion behaviors during the development of tumor drug resistance are all changed [33]. The co-culture of MM plasma cells and BMSCs could activate adhesion molecules to secrete the chemokines and cytokines, which promoting the migration, MM cell growth and drug resistance [34–36]. Johannes M et al found that interference of CXCL12 and CXCR4 could functionally interfered with MM chemotaxis to the bone marrow (BM), which led to the resensitization of MM cells to drugs by reversing the CAM-DR in MM [36]. In this study, we also construct the CAM-DR model with the co-culture of BMSCs and MM cells. ANXA7 overexpression further promoted the expression of cell adhesion molecules in the co-culture of BMSCs and MM cells to become more insusceptible to bortezomib, which was reversed by ANXA7 interference. Furthermore, CDC5L interference could alleviated the effect of ANXA7 overexpression on the expression of cell adhesion molecules to make the MM cells become more susceptible to bortezomib.

In conclusion, ANXA7 expression was increased in serum of MM patients and expression of ANXA7 and CDC5L was also increased in MM cells. ANXA7 overexpression promoted the proliferation and cell cycle of MM cells which was inhibited by ANXA7 interference. And, the effect of ANXA7 overexpression on the proliferation and cell cycle of MM cells could be reduced by CDC5L interference. Furthermore, ANXA7 overexpression promoted the CAM-DR in MM cells to make MM cells become more insusceptible to bortezomib while inhibited by ANXA7 interference. CDC5L interference inhibited the CAM-DR in MM cells to alleviate the promotion effects of ANXA7 overexpression on CAM-DR to make MM cells become more susceptible to bortezomib. ANXA7 was also demonstrated to be combined with CDC5L. Therefore, ANXA7 interference could promote cell cycle arrest in G2/M phase through CDC5L to inhibit proliferation of MM cells and reduce CAM-DR.

## MATERIALS AND METHODS

### Human serum specimens

The serum specimens were provided by 15 MM patients and 15 healthy donors from the Affiliated Hospital of Nantong University between January 2019 and October 2019. None of the patients had received treatment before. Everyone who took part in this study should sign the informed consent. The study was approved by the Ethics Committee of the Affiliated Hospital of Nantong University. The obtained serum specimens were centrifuged and preserved under -80 °C condition.

### Cell culture and bortezomib treatment

Human MM cell lines (U266, OPM-2 and RPMI-8226 cells), human bone marrow stromal cell HS-5 and human BMSC cells were brought from the American Type Culture Collection. The condition of cell culture was that RPMI 1640 medium with 10% fetal bovine serum (FBS), streptomycin (100 μg/mL), and penicillin (100 U/mL) (Invitrogen) and incubator with 5% CO_2_ at 37 °C. Bortezomib at 4nM treated the cells for 48 h.

### RT-qPCR analysis

The total RNA of all cells was extracted by Trizol Reagent (Thermo Fisher Scientific, Inc., USA) in accordance with the reagent instructions, and the RNA concentration was determined by Nanodrop 2000. According to the instructions of GoScriptTM Reverse Transcription System kits (Promega (Beijing) Biotech Co., Ltd, China), 1 μg total RNA was reverse-transcribed into cDNA. The reaction condition of RTFQ-PCR is that 95 °C for 30 s, followed by 40 cycles of 95 °C for 5 sec and 60 °C for 30 sec. GAPDH was an internal control and mRNA expression was quantitatively analyzed by 2^-ΔΔCq^ method.

### Western blot analysis

Total proteins were extracted from all cells and BCA method was used to detect protein concentration. The 10% SDS-PAGE was prepared and the gel electrophoresis board was put into the electrophoresis tank. Then, protein samples were subjected to SDS-PAGE electrophoresis. After electrophoresis, the protein samples were transferred to nitrocellulose membrane, which was transferred at the current of 95 mA for 3 h. After incubating in a blocking fluid containing skimmed milk powder for 4 h, membrane was incubated with ANXA7 (ab197586; Abcam; dilution, 1:2000), cell nuclear antigen (PCNA) (ab92552; Abcam; dilution, 1:2000), KI67 (ab16667; Abcam; dilution, 1:1000), cyclin dependent kinase 1 (CDK1) (ab32094; Abcam; dilution, 1:2000), cyclinB1 (ab181593; Abcam; dilution, 1:2000) and CDC5L (ab129114; Abcam; dilution, 1:2000) overnight at 4 °C. The next day, membrane was washed with TBST buffer containing 0.1% Tween20 for four times and incubated with goat anti-rabbit IgG-HRP second antibody (ab6721; Abcam; dilution, 1:2000) at room temperature for 1 h. After the washing of TBS, membrane was disposed with ECL and photographed with a Vilber Lourmat in the darkroom. Image J analysis system was used to analyze the gray values of each band.

### Cell transfection

U266 and RPMI-8226 cells were seeded in RPMI 1640 medium. When cells grow to 80%, the plasmids (overexpression-NC, overexpression-ANXA7, shRNA-NC, shRNA-ANXA7-1/2 and shRNA-CDC5L-1/2) (RiboBio Co., LTD, Guangzhou, China) were transfected into U266 and RPMI-8226 cells with Lipofectamine® 2000 reagent (Invitrogen, USA). No treatment was performed on the control group.

### CCK-8 assay

After cell transfection for 24 h, each hole of 96-well plates was added with 10 μL CCK-8 solution. The final solution volume in each hole was added to 100 μL with medium. At 24 h, 48 h and 72 h after cell transfection, the optical density value (OD value) was measured at the wavelength of 450 nm with a multifunctional microplate device.

### Colony formation assay

After cell transfection for 24h, the culture medium of U266 and RPMI-8226 cells was changed every two days. When colonies were visible to the naked eye, methanol was used to fix U266 and RPMI-8226 cells for 15 min and crystal violet was used to stain U266 and RPMI-8226 cells for 20 min. The residual dye solution was gently washed with double steam water, natural drying.

### Flow cytometry analysis

After cell transfection and bortezomib treatment for 48 h, a certain number of transfected U266 and RPMI-8226 cells were collected. The supernatant was discarded after 5min of centrifugation at 1000 r/min. 2 mL precooled anhydrous ethanol was added to fix the U266 and RPMI-8226 cells. After centrifugation at 1000 r/min for 5min, the ethanol was removed. The phosphate buffer saline (PBS) washed the U266 and RPMI-8226 cells twice and was removed after 5min of centrifugation at 1000 r/min. 10 μL Annexin-FITC and 10 μL 20 μg/mL propidium iodide (PI) was added to cells, which was incubated in darkness at room temperature. After incubation of 30min, the ratio of cells in G0/G1, S or G2/M phases to total cells was analysed by flow cytometry.

### TUNEL assay

The experiment was conducted by TUNEL detection kit (Beijing ZhongShan Biotechnology Company) according to its instruction. The main steps were as follows: the sample slide was digested by protease K and then treated with TdT and Biotin-dUTP. Sealed by the sealing liquid, sample slide was orderly treated with streptavidin-HRP working liquid and DAB color reagent. The color was observed and counted under the light microscope.

### ELISA assay

After cell transfection for 24 h, the expression of cell adhesion molecules (CD44, ICAM1 and VCAM1) in U266 and RPMI-8226 cells was assessed by the corresponding ELISA assay kits according to the manufacturer's protocols. The absorbance value was recorded at 450 nm using a microplate reader.

### Co-immunoprecipitation method

Protein from multiple myeloma cells was extracted from RIPA lysate containing protease inhibitor. The whole cell extract was divided into three parts, including IgG group, IP (co-immunoprecipitation) group and Input group. Rabbit anti-ANXA-7 antibody or rabbit anti-CDC5L antibody were added to the IP group for 4 h at 4 °C, while the corresponding homologous irrelevant antibody (rabbit polyclonal anti-IgG antibody) was added to the IgG group for 4 h at 4 °C. The protein A/G agarose beads were added into the IgG group and IP group, which were incubated for 1 h at 4 °C. RIPA lysate was used to wash the magnetic beads and the protein loading buffer re-suspend the magnetic beads to obtain the precipitated proteins. After centrifugation at 4000 rpm, the supernatant was collected and detected by Western blot analysis.

### Statistical analysis

The statistical analysis was conducted by SPSS 19.0 software. The experimental data were represented as mean±standard deviation (SD). Student t test (between two groups) or Dunnett’s-test (among three and above groups) was applied to analyse the significant difference in different groups. The value of P < 0.05 indicated the statistical significance.
